# Cancer-associated fibroblasts provide a suitable microenvironment for tumor development and progression in oral tongue squamous cancer

**DOI:** 10.1186/s12967-015-0551-8

**Published:** 2015-06-21

**Authors:** Huan Li, Ji Zhang, Shu-Wei Chen, Lu-lu Liu, Lei Li, Fan Gao, Shi-Min Zhuang, Li-ping Wang, Yan Li, Ming Song

**Affiliations:** State Key Laboratory of Oncology in South China, Collaborative Innovation Center of Cancer Medicine, 651 Dongfeng Dong Road, Guangzhou, 510060 People’s Republic of China; Department of Intensive Care, Sun Yat-sen University Cancer Center, Guangzhou, People’s Republic of China; Department of Head and Neck Surgery, Sun Yat-sen University Cancer Center, Guangzhou, People’s Republic of China; Department of Experimental Research, Sun Yat-sen University Cancer Center, Guangzhou, People’s Republic of China; Department of Otolaryngology-Head & Neck Surgery, The Third Affiliated Hospital of Sun Yat-sen University, Guangzhou, People’s Republic of China; The People’s Hospital of Bao’an District Shenzhen, Shenzhen, People’s Republic of China

**Keywords:** Oral tongue squamous cell cancer, Microenvironment, Cancer-associated fibroblast, Progression

## Abstract

**Background:**

Oral tongue squamous cell carcinoma (OTSCC) is still associated with a poor prognosis due to local recurrence and metastasis. Cancer-associated fibroblasts (CAFs) play an important role in the complex processes of cancer stroma interaction and tumorigenesis. This study aims to determine the role of CAFs in the development and progression of OTSCC.

**Methods:**

Immunohistochemistry was performed to evaluate the frequency and distribution of CAFs in 178 paraffin specimens from patients with OTSCC. Immunofluorescence, a cell proliferation assay, flow cytometry, migration and invasion assays and western blot analysis were used to study the effects of CAFs and the corresponding conditioned medium (CM) on the proliferation and invasion of OTSCC cell lines.

**Results:**

Statistical analysis showed a strong correlation between the frequency and distribution of CAFs and the clinicopathological characteristics of patients with cN0 OTSCC, including pathological stage (*P* = 0.001), T classification (*P* = 0.001), and N classification (*P* = 0.009). Survival analysis demonstrated a negative correlation of the frequency and distribution of CAFs with the overall survival and disease-free survival of patients with cN0 tongue squamous cell cancer (*P* = 0.009, 0.002, respectively); Cox regression analysis showed that the presence of CAFs (relative risk: 2.113, CI 1.461–3.015, *P* = 0.023) is an independent prognostic factor. A functional study demonstrated that CAFs and CM from CAFs could promote the growth, proliferation, mobility, invasion and even Epithelial Mesenchymal Transition (EMT) of OTSCC cells compared with NFs and CM from NFs.

**Conclusions:**

CAFs were an independent prognostic factor for patients with OTSCC. Compared with NFs, CAFs and their CM have the ability to promote the growth, proliferation, metastasis and even EMT of OTSCC cells.

**Electronic supplementary material:**

The online version of this article (doi:10.1186/s12967-015-0551-8) contains supplementary material, which is available to authorized users.

## Background

Oral cancer is the tenth most commonly diagnosed cancer in men worldwide and accounts for 263,900 cases worldwide [1]. The tongue is the most cancer-prone intraoral site in most populations that have been studied, and the most common pathological type of oral tongue cancer is squamous cell cancer [2]. Although intensive efforts have been made in primary prevention and in the improvement of therapy, the morbidity and mortality rates for oral tongue cancer remain steady and high and have even continued to rise in some developing countries. Due to its highly invasive nature, oral tongue squamous cancer frequently leads to severe defects in speech, mastication and deglutition, as well as cancer-related death. During the last three decades, the long-term survival rate for patients with oral tongue cancer has not improved substantially, and the tongue remains among the worst sites for all cancers with respect to survival [3].

Cancer has long been considered a cell-autonomous process in which progressive genetic and epigenetic alterations transform cells independently of the external milieu. Substantial evidence indicates that the interactions between tumor cells and their microenvironment are of great importance in the development and progression of cancer. Cancer cells may alter the surrounding cancer stroma and, in turn, cancer stromal cells and cytokines may promote cancer progression and the acquisition of invasive properties [4, 5].

According to new data that have accrued, the cancer stroma can be described as an active microenvironment that modulates the biology of the carcinoma including cancer stem cells [6]. The cancer stromal portion of a carcinoma is formed by the interaction of cancer stromal cells and cytokines in the extracellular matrix, which is produced by fibroblasts, macrophages and other inflammatory cells as well as blood/lymphatic capillaries [7]. Recent progress in cancer biology has markedly changed our view with regards to the functional significance of the cancer stroma. A sub-population of cells in the cancer stroma, with a myofibroblast-like phenotype, has been termed cancer-associated fibroblasts (CAFs) [8, 9]. In the cancer stroma, CAFs undergo changes in protein expression that represent an ‘activated’ myofibroblastic phenotype, which typically involves the up-regulation of markers such as α-smooth muscle actin (α-SMA) [10, 11]. By imitating activated ‘wound repair’ fibroblasts, CAFs are thought to promote the progression of carcinoma via the stimulation of epithelial cell growth, migration and invasion [5, 12, 13].

CAFs represent an additional histopathologic feature and are of great importance in the complex cancer stroma. It was reported that CAFs are derived from normal cancer stromal fibroblasts under the direct impact of cancer cell-derived cytokines and function to further facilitate local and distant migration as well as aid in the suppression of the host immune response [14–16]. In addition, emerging evidence has also shown that the malignant epithelial cells themselves may be a significant source of CAFs [14, 17]. This phenomenon is termed epithelial–mesenchymal transition (EMT), during which epithelial cells lose their specific markers and acquire the characteristics of mesenchymal cells [18, 19].

A recent study on human tongue carcinogenesis showed that CAFs emerged concomitantly with the development of carcinomas, but were practically absent in dysplastic, pre-malignant lesions [20]. CAFs have a distinctive phenotype compared with quiescent fibroblasts in differentiated adult tissue, but the morphologic and functional differences are not completely understood. Gene expression profiling by cDNA microarray technology can vastly aid in the characterization of CAFs that are isolated from a broad range of solid tumors, including pancreatic [21], colon [22], breast [23], and basal cell cancers [24].

Studies of different cancer types have shown that CAFs are located in the vicinity of tumor cells and are able to enhance tumor growth through the secretion of growth factors (e.g., transforming growth factor-μ), matrix degrading enzymes [e.g., matrix metalloproteinases (MMPs)], and angiogenic factors (e.g., vascular endothelial growth factor) [25–30]. This study aims to determine the role of CAFs in the development and progression in oral tongue squamous cell carcinoma (OTSCC) and to reveal the characteristics of gene expression in CAFs compared with NFs.

## Methods

### Patients, tissue specimens and cell lines

The specimens were used after prior written consent was obtained from the patients as well as after the approval of the Institutional Research Ethics Committee of the Sun Yat-sen University Cancer Center. A total of 178 tissue specimens were removed from patients with cN0 oral tongue cancer, none of whom had received radiotherapy or chemotherapy prior to surgery. Sixty years of age was selected as the cut-off point because it is the mean age of the overall patient population with oral SCC [31]. Patient progress was followed for 78.3 ± 42.1 months (mean ± SD). The clinical characteristics of this patient cohort are summarized in Additional file 1: Table S1.

Eight pairs of primary OTSCC tissues and surrounding nontumor oral tongue tissues were collected at the time of surgical resection at the Cancer Center of Sun Yat-sen University. None of the patients received adjuvant therapy prior to surgery.

Four human oral tongue cancer cell lines were purchased from the American Type Culture Collection (SCC-25 and CAL-27) and the Cell Bank of Type Culture Collection of the Chinese Academy of Sciences (TSCCa and Tca-8113). All cells were grown in 5% CO_2_ in a humidified atmosphere at 37°C.

### Immunohistochemistry

With regards to the double immunostaining, one additional section from each case was subjected to a staining protocol that combined two markers: α-SMA and Ki-67. Sections were pre-treated with an EDTA buffer at pH 9 and heated in a pressure cooker. Sections were then exposed to a mixture of antibodies against Ki-67 (polyclonal, 1:50, Dako, Denmark) and α-SMA (clone 1A4, 1:100, Dako, Denmark) and incubated for 1 h at room temperature. Then, the sections were treated with MACH 2 double stain polymer detection kit #2 (mouse-HRP + rabbit) alkaline phosphatase (ALP)) (Biocare Medical, Concord, CA, USA), followed by 3,3′-diamino-benzidine (DAB), Vulcan Fast Red chromagen kit 2 (Biocare Medical, USA), and denaturing solutions A and B (Biocare Medical, USA). Afterwards, the sections were treated with MACH2 polymer-ALP conjugate (goat antimouse polymer-ALP secondary antibody) (Biocare Medical, USA). This was followed by treatment with Ferangi Blue chromagen system (Biocare Medical, USA). Finally, the slides were covered by GVA mounting medium (Zymed, San Francisco, CA, USA). As a result, the nuclear staining of Ki-67 was visualized as a red fuchsin color, while the cytoplasmic staining of α-SMA was brown. Special emphasis was placed upon the assessment of the relationships between the tumor cells and the adjacent CAFs at the tumor–host interface.

The immunohistochemically stained slides were examined for the pattern of distribution of CAFs throughout the entire cancer stroma [32].

**Figure 1:**
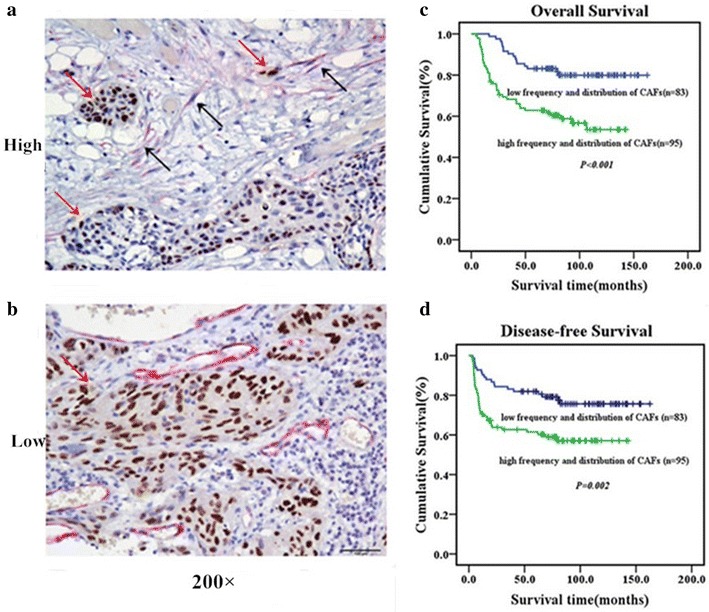
Representative double immunohistochemical images of cN0 oral tongue cancer tissue specimens, which indicate high frequency and distribution of CAFs (**a**) and low frequency and distribution of CAFs (**b**). Tumor cell nuclei are stained a brown with the Ki-67 antibody, whereas CAFs are stained a fuchsin color in the cytoplasm by the α-SMA antibody . Magnification is ×200 (**a**, **b**). The frequency and distribution of CAFs affect overall survival and disease-free survival. Kaplan–Meier curves with univariate analysis (log-rank) for patients with cN0 oral tongue cancer with a high frequency and distribution of CAFs (n = 95) versus a low frequency and distribution of CAFs (n = 83) with respect to overall survival (**c**) and disease-free survival (**d**).

Quantitatively, the number of CAFs was assessed on a 5-scale scoring system: 0, devoid of CAFs; 0.5, a few CAFs with a spindle-shaped morphology that adhered closely to the periphery of the SCC islands/nests; 1, CAFs surrounded the tumor in a few concentric layers in several foci; 2, CAFs with both a spindle-shaped and a plump morphology were observed in many areas of the tumor; 3, similar to a score of “2”, but CAFs were exceptionally abundant throughout the section and occasionally exceeded the carcinomatous component (Figure 1). For the purpose of statistical analysis, cases with low scores (0, 0.5, and 1) were combined and compared with cases with high scores (2 and 3).

### Isolation of fibroblasts

Cancer tissue and its paired normal oral tongue tissue were cut into the smallest pieces possible in sterile PBS solution and were then digested with collagenase (0.1% collagenase type IV, Sigma) at 37°C for 30 min. The suspension was filtered through a 20-μm stainless steel wire mesh to collect a single-cell suspension. The filtrate was centrifuged at 1,500 rpm for 5 min and washed twice with DMEM before the cells were plated on 6-cm tissue culture dishes in 5 mL DMEM supplemented with 20% fetal bovine serum. After 30 min in culture at 37°C, the nonadherent cells (mainly tumor cells) were removed to obtain a population of pure fibroblasts because the adhesion time needed for fibroblasts is much shorter (20–30 min) than that for tumor cells (usually >1 h). The adherent fibroblasts were sub-cultured for further study.

### Immunofluorescence

Cells were grown on gelatin-coated coverslips for 24 h and then fixed in 4% paraformaldehyde at room temperature for 20 min. Cells were incubated with PBS-B solution at 37°C for 30 min to block nonspecific interactions and were then incubated overnight at 4°C with various ready-to-use primary antibodies against fibronectin, E-cadherin, α-SMA. After several washes in PBS, the cells were incubated with optimal concentrations of FITC-labeled secondary antibody (Dako) at room temperature for 1 h. Antifade 4′,6-diamidino-2-phenylindole solution was added, and images were obtained.

### Detection of DNA content by flow cytometry

In regard to the flow cytometric analysis, 1–2 × 10^6^ cells were fixed in 70% ethanol and stained with propidium iodide. The DNA content was then analyzed with a Cytomics FC device (Beckman Coulter). Finally, the cell cycle profiles were analyzed with Modfit LT2.0. To determine the ploidy pattern of the tested cells (CAFs, NFs, and cancer cells), a DNA index was calculated based as the ratio of the G1 peak channel of the tested cells to the G1 peak channel of normal peripheral blood lymphocytes. All experiments were performed independently and in triplicate.

### Cell growth assay

The cell growth rates of fibroblasts derived from OTSCC tissue and normal tissue were determined by 2,3-Bis-(2-methoxy-4-nitro-5-sulfophenyl)-2H-tetrazolium-5-carboxanilide (XTT) assay. Cells were seeded into 96-well plates at a density of 1 × 10^3^ per well. The XTT kit (Sigma) was used according to the manufacturer’s instructions. All experiments were performed independently and in triplicate.

### Effect of fibroblasts on the growth and migration of OTSCC cells

A 3-D culture was performed to examine the effect of fibroblasts on the growth and migration of OTSCC cells. CAFs, NFs, TSCCa or CAL-27 cells were seeded into 24-well plates that were coated with 50 μl BioCoat Matrigel at a density of 5.0 × 10^3^ per well. CAFs or NFs were added to eight plates with TSCCa or CAL-27 cells plated at a density of 2.0 × 10^3^ per well. We observed the aggregation of the cells after 1 week of culture in 5% CO_2_ in a humidified atmosphere at 37°C. All experiments were performed independently and in triplicate.

### Preparation of the conditioned media

OTSCC cells (TSCCa and CAL-27) and fibroblasts (CAFs and NFs) were seeded into T75 culture flasks and grown in normal growth media (10 mL of DMEM with 10% fetal bovine serum) for 48 h until the cells were approximately 80% confluent. The culture medium was then collected from each flask and centrifuged at 1,000 rpm for 30 min, and the supernatant was collected as conditioned medium (CM) for further study.

### Effect of CM from fibroblasts on the growth of OTSCC cells

To examine the effect of CM from fibroblasts on the growth of OTSCC cells, TSCCa or CAL-27 cells were seeded into 96-well plates at a density of 1.5 × 10^3^ per well and cultured in CM from CAFs and NFs. CM from TSCCa or CAL-27 cells was used as a control. The cell growth rate was determined by XTT assay as described previously. Independent experiments were performed in triplicate.

### Effect of CM from fibroblasts on the migration of ESCC cells

Cell mobility was assessed by a scratch wound-healing assay. TSCCa or CAL-27 cells were grown to confluence in a six-well plate with normal growth medium. The cell monolayer was mechanically scratched with a sterile pipette tip. Subsequently, the cells were incubated with CM from CAFs or NFs instead of normal growth medium. CM from TSCCa or CAL-27 cells was used as a control. Cell mobility in terms of wound closure was measured by imaging three random fields at the time points 0, 24 and 48 h.

### Effect of CM from fibroblasts on the invasiveness of ESCC cells

An invasion assay was performed with a BioCoat Matrigel Invasion Chamber (BD Biosciences) according to the manufacturer’s instructions. Briefly, TSCCa or CAL-27 cells (2.5 × 10^5^) were placed in the upper compartment of each chamber. The lower compartment was filled with CM from TSCCa cells (or CAL-27 cells), CAFs, or NFs. After 24 h, cancer cells that had penetrated the Matrigel-coated membrane and passed into the lower compartment were stained and counted. Independent experiments were performed in triplicate.

### Effect of CM from fibroblasts on the EMT of OTSCC cells

To examine the effect of CM from fibroblasts on the EMT of OTSCC cells, TSCCa or CAL-27 cells were seeded into 96-well plates at a density of 1.5 × 10^3^ per well and cultured in CM from CAFs or NFs. CM from TSCCa or CAL-27 cells was used as a control. Total cell protein was collected from each plate, and the expression of epithelial markers(α-catenin, β-catenin, E-cadherin)and mesenchymal markers (α-SMA, vimentin) was detected by ECL prime western blotting detection reagent (Amersham) according to the manufacturer’s instructions. β-actin was used as a loading control.

### Statistical analysis

Statistical analysis was conducted with SPSS standard version 17.0 software. Data are expressed as the mean ± SD from at least three independent determinations. The significance of the differences was analyzed using Student’s t tests. Differences were considered significant at *P* < 0.05.

## Result

### The relationship between the frequency and distribution of CAFs and the clinicopathological characteristics and prognosis of patients with OTSCC

We further investigated the link between the frequency and distribution of CAFs and the clinicopathological characteristics of patients with oral tongue cancer using a panel of 178 paraffin-embedded, archived oral tongue cancer specimens. The frequency and distribution of CAFs was analyzed by double immunohistochemical staining with an anti-α-SMA antibody and an anti-Ki67 antibody. High frequency and distribution of CAFs were detected in 95 samples (53.4%) and low frequency and distribution were observed in 83 tumor samples (46.6%) (Figure 1a).

Statistical analysis showed a correlation between the frequency and distribution of CAFs as determined by immunohistochemistry and the clinicopathological characteristics of cN0 oral tongue cancer, including pathologic stage (*P* = 0.001), T classification (*P* = 0.004), N classification (*P* = 0.024), recurrence (*P* = 0.010) and vital status (*P* = 0.001). In contrast, the frequency and distribution of CAFs did not correlate with age or gender (Additional file 1: Table S1).

A survival analysis showed a clear negative correlation between the frequency and the distribution of CAFs with the overall survival and the disease-free survival of patients with cN0 tongue squamous cell cancer (*P* < 0.001, *P* = 0.002, respectively) (Figure 1b). A Cox regression analysis revealed that only N classification (relative risk, 2.479, CI 1.486–4.571, *P* < 0.001) and the frequency and distribution of CAFs (relative risk: 2.113, CI 1.461–3.015, *P* = 0.023) were independent prognostic factors for poor overall survival.

### Isolation of CAFs and NFs

**Figure 2:**
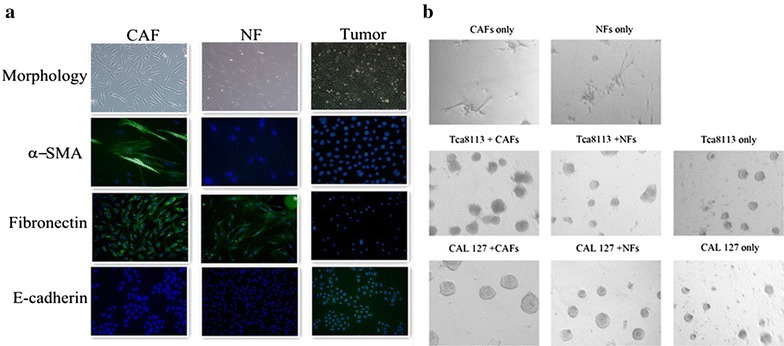
Representative cell morphology of CAFs, NFs, and tumor cells (OTSCC). Immunofluorescence was used to distinguish CAFs, NFs and OTSCC cells with antibodies that target α-SMA, fibronectin, and E-cadherin (**a**). Neither CAFs nor NFs grow or aggregate in matigel. Tca8113and CAL-27co-cultured with CAFs grow and aggregate better than that with NFs (**b**).

CAFs and their paired NFs were successfully isolated from eight primary OTSCCs by short-term primary culture in DMEM supplemented with 10% fetal bovine Serum. To confirm that CAFs derived from tumor tissue were part of a population of pure fibroblasts without tumor cell contamination, several cell markers including the epithelial cell marker E-cadherin and the fibroblast marker fibronectin, were used to distinguish fibroblasts from tumor cells. In addition, the CAF-specific marker α-SMA was also used. The immunohistochemistry results demonstrated that the CAFs expressed the fibroblast-specific marker fibronectin as well as the CAF-specific marker α-SMA. However, the NFs expressed only the fibroblast-specific marker fibronectin, and OTSCCs expressed only the epithelial cell marker E-cadherin (Figure 2a). The 3-D cell culture showed that OTSCCs that were co-cultured with CAFs grew and aggregated more quickly than when they were co-cultured with NFs (Figure 2b).

### CAFs demonstrate a stronger ability to promote the growth, proliferation, and mobility of OTSCC cells compared with NFs

**Figure 3:**
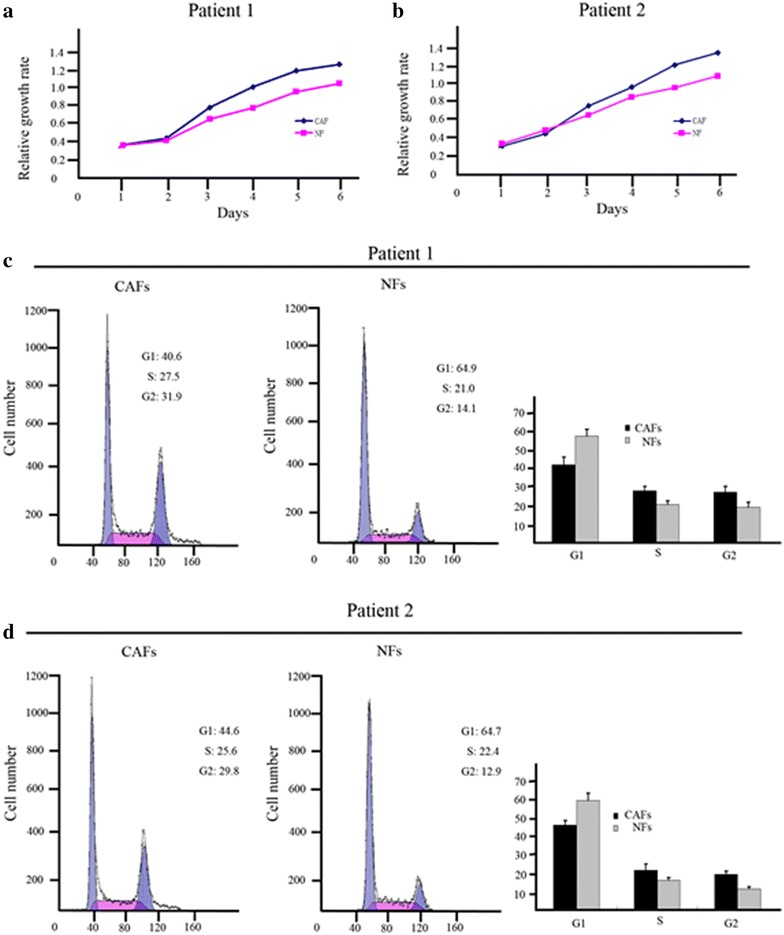
CAFs grow faster than NFs. XTT assay was used to compare cell growth rates between CAFs and NFs (**a**, **b**). Two examples of DNA content comparison between CAFs, NFs by flow cytometry. Flow cytometry histogram shows that the percentages of cells in S and G2-M phases were significantly higher in CAFs compared with NFs (**c**, **d**).

The cell growth assay revealed that the cell growth rate of CAFs was significantly higher than that of NFs under the same culture conditions (*P* < 0.05; Figure 3a, b). A flow cytometric analysis showed that the percentage of cells in the S and G2-M phases was significantly higher in CAFs compared with NFs (*P* < 0.05), which suggests that CAFs had a stronger capacity for proliferation than their paired NF counterparts (Figure 3c, d).

### Effect of CM from CAFs

**Figure 4:**
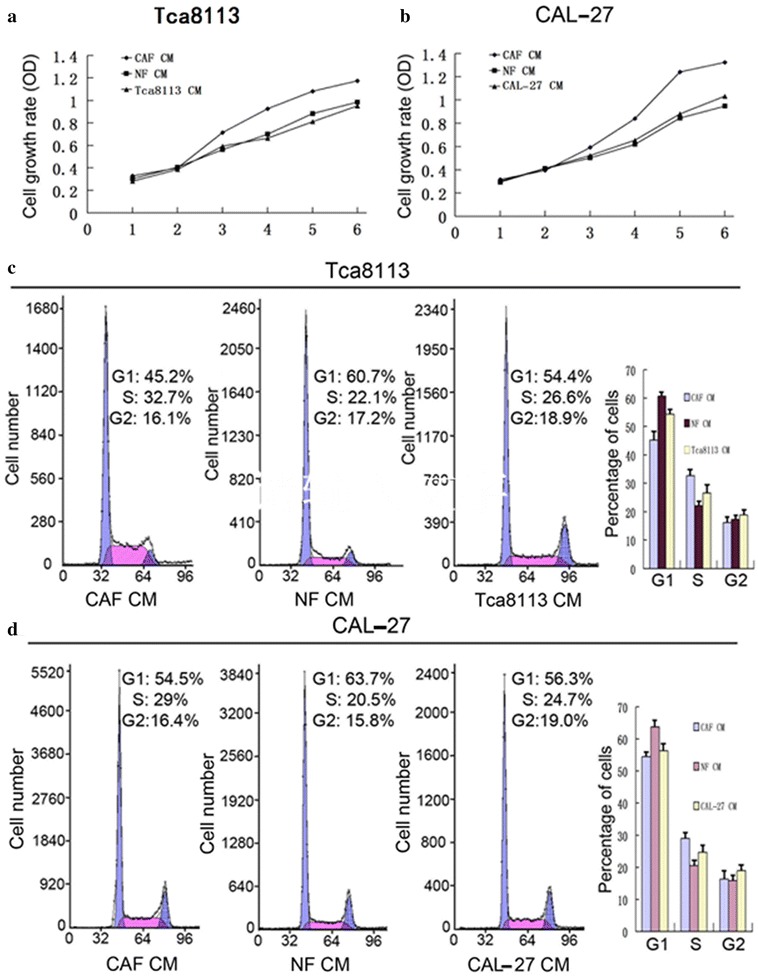
Tca8113 and CAL-27 in CAFs CM grow faster than in NFs CM. XTT assay was used to compare cell growth rates between Tca8113or CAL-27 in CAFs and NFs CM (**a**, **b**). Two examples of DNA content comparison between Tca8113 and CAL-27 in CAFs CM, NFs CM and its own CM by flow cytometry. Flow cytometry histogram shows that the percentages of cells in S and G2-M phases were significantly higher in CAFs CM compared with NFs and its own CM (**c**, **d**).

**Figure 5:**
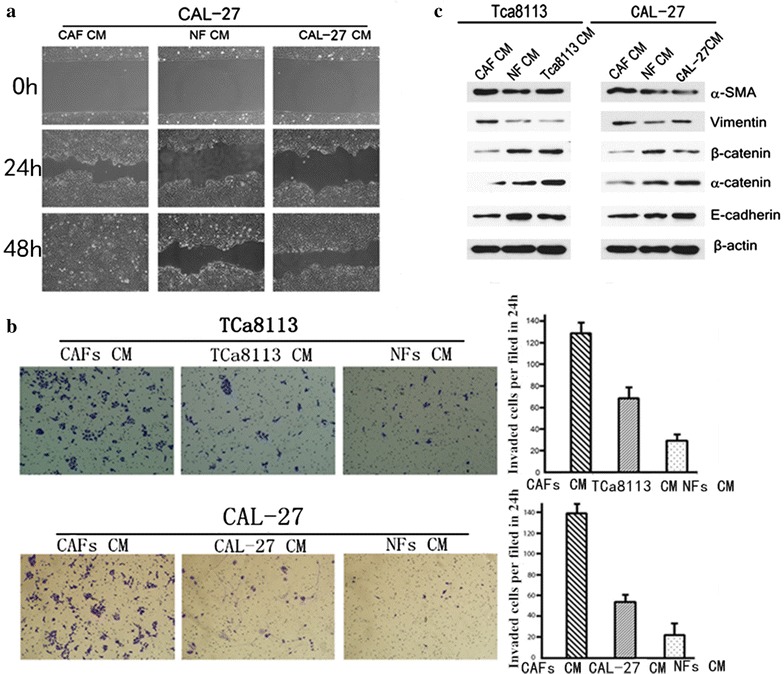
CAFs promote cell mobility and invasion. **a** The effect of CAFs on cell migration was determined by wound-healing assay. During a period of 48 h, the spreading speed of CAL-27 in CAFs CM along the wound edge was faster than that in NFs and its own CM. **b** Representative images showed that Tca8113 and CAL-27 in CAFs, NFs and its own CM invaded through the matrigel. The number of invaded tumour cells was quantified in the *right panel*. Columns, mean of triplicate experiments, *P* < 0.01. **c** Expressions of epithelial markers α-catenin, β-catenin and E-cadherin and mesenchymal markers α-SMA and vimentin were compared by western blotting analysis between Tca8113 and CAL-27 in CAFs, NFs and its own CM. β-actin was used as a loading control.

The cell growth assay showed that OTSCC cells which are cultured in CM from CAFs grow faster than when they are cultured in CM from NFs (Figure 4a, b). A flow cytometric analysis revealed that the S/G2 peak was more common in OTSCC cells grown in CM from CAFs compared with OTSCC cells grown in CM from NFs (Figure 4c, d). A cell wound healing assay showed that OTSCC cells grown in CM from CAFs have greater capacity for proliferation and mobility compared with OTSCC cells grown in CM from NFs (Figure 5a). A Transwell invasion assay both showed that OTSCC cells growing in CM from CAFs have greater mobility and a greater capacity for invasion and proliferation compared with OTSCC cells growing in CM from NFs (Figure 5b). A western blot assay showed that OTSCC cells grown in CM from CAFs expressed lower levels of epithelial markers (α-catenin, β-catenin, E-cadherin) but expressed higher levels of mesenchymal markers (α-SMA, vimentin) compared with OTSCC cells grown in CM from NFs (Figure 5c).

## Discussion

The tongue is the most cancer-prone intraoral site in most populations that have been studied and remains among the worst sites for all cancers in terms of survival [3]. The most common pathological type of oral tongue cancer is squamous cell carcinoma [2]. In clinical practice, head and neck surgeons depend primarily on the TNM classification system to plan treatment strategies, and no consensus exists on the optimal treatment of patients with cN0 oral tongue cancer [33]. However, the TNM system is not sufficiently reliable for the prediction of clinical outcome or for the establishment of detailed information on the biological characteristics of a malignancy [34]. The poor prognosis of oral tongue cancer is mainly a consequence of its unusual histological makeup, which means that this cancer type is poorly equipped to resist invasion and metastasis [35]. Attempts have been made to elucidate the underlying mechanism of the development of oral tongue carcinoma based on the characteristics of the cancer cells themselves; however, a clear cancer-associated gene or molecular machinery of the pathogenesis has not yet been established. With the exception of the traditional therapeutic program, including surgery, radiotherapy and chemotherapy, drugs that effectively target genes have not been adequately developed.

Cancer, a widespread major public health threat, is believed to be a multistep process with numerous accumulated genetic changes that occur during disease development and progression. Recent findings that tumors do not act alone, but rather, are surrounded by a malignant microenvironment composed of the tumor-associated cancer stroma, which is created by and acts for the tumor itself, may lead to a reassessment of this concept. CAFs are now recognized as the main effectors of tumor needs in terms of angiogenesis, collagen breakdown, further invasion and suppression of the host immune response [15–17]. Marilena Vered et al. performed a triple immunostaining assay in tongue squamous cell cancer with E-cadherin, Ki-67 and α-smooth muscle actin to identify carcinoma cells undergoing epithelial–mesenchymal transition. They also used a Kaplan–Meier survival analysis with univariate and Cox multivariate regression models with stepwise forward selection, and Fisher’s exact tests to confirm that local recurrence and overall survival were negatively influenced by abundance of cancer stromal myofibroblasts [32]. We used a panel of 178 paraffin-embedded, archived oral tongue cancer specimens to perform double immunohistochemical staining with an anti-α-SMA antibody and an anti-Ki67 antibody. Statistical analysis showed a correlation between the frequency and distribution of CAFs, as determined by immunohistochemistry and the clinicopathological characteristics of patients with cN0 oral tongue cancer, including pathologic stage, T classification, N classification, recurrence and vital status. A survival analysis showed a clear negative correlation of the frequency and distribution of CAFs with overall survival and disease-free survival in patients with cN0 tongue squamous cell cancer. These results are in agreement with those of previous studies.

One of the current evidence-based theories is that at least some of the CAFs actually represent malignant cells that have undergone epithelial–mesenchymal transformation (EMT) [36–38]. In our recent study, by WB, we were able to demonstrate abnormal expression of key factors of tumor cells undergoing EMT in cases of human tongue carcinoma. These processes might provide a reasonable explanation for our observation that an abundance of CAFs were found to be significantly associated with disease recurrence and patient survival. The present results are in agreement with those of studies on CAFs in various types of carcinomas, including pancreas [21], colon [22], breast [23], basal cell cancer [24] and gastric [39].

In the present study, we isolated and characterized CAFs from oral tongue squamous cancer tissue. The cell growth rate of CAFs was significantly higher (*P* < 0.05) than that of paired fibroblasts from nontumor tissue. Flow cytometry revealed that the percentage of proliferating cells (S and G2-M phases) was also significantly higher in CAFs than in NFs (P < 0.05). A 3D co-culture model compared CAFs and NFs co-cultured with OTSCC lines. This provided a valuable tool to dissect the complex interactions between these cell populations to understand the factors that determine the behavior of breast cancer. These results showed the differences between CAFs and NFs not only in their morphologic appearance but also in their biological behavior, which may be due to genetic mutations.

Paracrine production of cytokines by CAFs has also been reported in multiple tumor types. High levels of serum FGF2 have been observed in individuals with small cell lung cancer and are associated with a poor prognosis [40]. This may be due to an FGF2-mediated cytoprotective effect, whereby the expression of antiapoptotic proteins is up-regulated, which promotes resistance to current anticancer treatments [41]. Increased paracrine expression of one or more of FGF1, 2, 4, 5, 8, and 18 has been found to promote tumor neoangiogenesis in preclinical models via the main endothelial FGFRs, FGFR1 and 2 [42]. Poor prognosis has been associated with neoangiogenesis in ovarian cancer and melanomas [43]. Previous studies have demonstrated that the potential mechanism that mediates tumorigenesis and progression includes PI3 K-Akt, MAPK, Wnt/beta-catenin and other signaling pathways. Based on our study results, we have enough evidence to hypothesize that some paracrine production of CAFs may be involved in some tumorigenic mechanism to achieve EMT.

## Conclusions

CAFs were an independent prognostic factor for patients with OTSCC. Compared with NFs, CAFs and their CM have the ability to promote the growth, proliferation, migration and even EMT of OTSCC cells.

## Additional file

Additional file 1:
**Table S1**. Correlation the frequency and distribution of CAFs and clinicopathologic characteristics of patients with clinically N0 oral tongue cancer.
