# Supplementary material for: Cancer-associated fibroblasts provide a suitable microenvironment for tumor development and progression in oral tongue squamous cancer
Source: J Transl Med. 2015 Jun 21;13:198. doi: 10.1186/s12967-015-0551-8 (PMC4475624; doi:10.1186/s12967-015-0551-8)
Supplement: Additional file 1: — Table S1. Correlation the frequency and distribution of CAFs and clinicopathologic characteristics of patients with clinically N0 oral tongue cancer. [file 12967_2015_551_MOESM1_ESM.doc]

**Table S1. Correlation the** **frequency and distribution of CAFs and clinicopathologic characteristics of patients with clinically N0 oral tongue cancer**

| **Characteristics** | ***n*** | ** α−SMA expression** | | **χ^2^ test *P*** |
| --- | --- | --- | --- | --- |
|  |  | **Low or none,**  **no. (%)** | **High,**  **no. (%)** | **(Fisher’s exact test *P*)** |
| **Gender** |  |  |  | **0.469** |
| Male | 106 | 46(43.40) | 60(56.60) |  |
| Female | 72 | 37(51.39) | 35(48.61) |  |
| **Age (years)** |  |  |  | **0.178** |
| <60 | 88 | 43(48.86) | 45(51.14) |  |
| ≥ 60 | 90 | 40(44.44) | 50(55.56) |  |
| **Pathologic stage** | |  |  | **0.001** |
| Ⅰ | 82 | 51(62.20) | 31(37.80) |  |
| Ⅱ | 64 | 26(40.62) | 38(59.38) |  |
| Ⅲ | 17 | 6(35.30) | 11(64.70) |  |
| Ⅳ | 15 | 0(0.00) | 15(100.0) |  |
| **T classification** | |  |  | **0.004** |
| T1 | 89 | 54(60.67) | 35(39.33) |  |
| T2 | 84 | 27(32.14) | 57(67.86) |  |
| T3 | 5 | 2(40.00) | 3(60.00) |  |
| **N classification** | |  |  | **0.024** |
| N0 | 148 | 77(52.03) | 71(47.97) |  |
| N1 | 15 | 6(40.00) | 9(60.00) |  |
| N2 | 15 | 0(0.00) | 15(100.0) |  |
| **Recurrence**  No | 118 | 67(56.78) | 51(43.22) | **0.010** |
| Yes | 60 | 16(26.67) | 44(73.33) |  |
| **Vital status (at follow-up)** | | |  | **0.001** |
| Alive | 121 | 74(61.16) | 47(38.84) |  |
| Dead | 57 | 9(15.79) | 48(84.21) |  |
